# Viability Prediction of *Ricinus cummunis* L. Seeds Using Multispectral Imaging

**DOI:** 10.3390/s150204592

**Published:** 2015-02-17

**Authors:** Merete Halkjær Olesen, Pejman Nikneshan, Santosh Shrestha, Ali Tadayyon, Lise Christina Deleuran, Birte Boelt, René Gislum

**Affiliations:** 1 Department of Agroecology, Science and Technology, Aarhus University, Slagelse 4200, Denmark; E-Mails: santosh.shrestha@agro.au.dk (S.S.); Lise.deleuran@agro.au.dk (L.C.D.); Birte.boelt@agro.au.dk (B.B.); Rene.gislum@agro.au.dk (R.G.); 2 Department of Agronomy, Shahrekord University, Shahrekord 88176-53849, Chaharmahal Bakhtiyari, Iran; E-Mails: Nikneshan@stu.sku.ac.ir (P.N.); tadayyon.sku@gmail.com (A.T.)

**Keywords:** multispectral imaging, castor seed, canonical discriminant analysis (CDA), viability, germination

## Abstract

The purpose of this study was to highlight the use of multispectral imaging in seed quality testing of castor seeds. Visually, 120 seeds were divided into three classes: yellow, grey and black seeds. Thereafter, images at 19 different wavelengths ranging from 375–970 nm were captured of all the seeds. Mean intensity for each single seed was extracted from the images, and a significant difference between the three colour classes was observed, with the best separation in the near-infrared wavelengths. A specified feature (RegionMSI mean) based on normalized canonical discriminant analysis, were employed and viable seeds were distinguished from dead seeds with 92% accuracy. The same model was tested on a validation set of seeds. These seeds were divided into two groups depending on germination ability, 241 were predicted as viable and expected to germinate and 59 were predicted as dead or non-germinated seeds. This validation of the model resulted in 96% correct classification of the seeds. The results illustrate how multispectral imaging technology can be employed for prediction of viable castor seeds, based on seed coat colour.

## 1. Introduction

Castor (*Ricinus communis* L.) is a significant non-edible oil crop, considered a vital industrial raw material. It is cultivated on 1.5 mio hectares worldwide with an annual seed production of 1.85 Mt and an average seed yield of 1235 kg·ha^−1^ [1]. The seeds are available at low cost and the plant is known to tolerate adverse weather conditions. Castor can be grown on marginal lands not suitable for food crops. These features combined make castor an attractive alternative biodiesel feedstock.

Seed quality is very important to optimize plant growth and yield production on farms. Rapid and uniform germination and subsequent seedling development and crop establishment are important factors influencing yield potential as the plant has limited ability to compensate for low plant densities [2,3]. Knowledge regarding seed vigour and viability is therefore significant to optimizing a future profitable production of castor.

Seeds within a castor plant mature sequentially both within a raceme and between racemes only a part of the seeds continuously being set will reach same maturity level by the end of the season [4]. The final harvest of a castor seed lot will thus result in seeds of different size, colour and physiological maturity and thus also possess different germination abilities [4,5]. Seed vigour and viability are influenced by environments during seed development, maturity levels at harvest and environments during storage [5]. Seed coat colour and the endosperm volume are known to be accurate indicators for assessing the seed developmental stage [6]. In previous studies of castor seeds, seed filling and inner morphology were evaluated by X-ray imaging, and a clear variation in germination percentage between the different seed groups, full or partially full seeds, was observed [7]. Seed weight correlated with germination capacity shows that the germination of light seeds was inferior to that of heavier seeds [5]. The seed coat colour was related to development status (weight, size, oil and nitrogen, phosphorus, potassium content), where seeds with dark seed coat appeared more developed, and seeds with clearest coat colour were smaller and less developed [8].

The tetrazolium test is a common biochemical test of seed viability. A viable seed should present staining in all those tissues whose viability is necessary for normal seedling development [9]. Correlation of a tetrazolium test (viability) with a germination test in sand plus seedling emergence in the field are efficient for evaluating castor seed quality [10] and therefore a potential indicator for quality determination.

Multispectral imaging is an emerging non-destructive technology in seed science, which integrates the conventional vision and spectroscopy technique to attain both spatial and spectral information from the target objects simultaneously. Multispectral imaging requires no sample pre-treatments, making it more suited for process monitoring and quality control. More importantly, this technique has a great potential to measure the multiple components by reflection from both visual (visual colour compounds) and near-infrared wavelengths (non-visual chemical compounds) at the same time for quality assurance. Vision and spectral technologies have shown promising results in different aspects of determing seed quality features such as fungal infection and germination in spinach [11–13] fungal infection in corn [14] and seed purity in rice [15,16].

The objective of this research was to evaluate the potential use of multispectral imaging as a rapid and non-destructive method in castor seed quality testing. We investigated the correlation between seed coat colour reflection and viability obtained by a tetrazolium test. Next, validations of the model of viable or dead seeds were tested, by sorting the seeds in accordance to a threshold value and correlate it with germination capacity.

## 2. Experimental Section

### 2.1. Seed Material

Castor seeds employed in the present research originated from field experiments in 2013 including four ecotypes: Esfahan, Naeen, Ahvaz and Arak, grown in two irrigation regimes (irrigation and non-irrigation). The ecotypes were from different regions of Iran and their names originated from their specific regions. Irrigation regimes were done based on a soil moisture curve [17]. These seeds were stored at 15 °C and 40% relative humidity after harvest.

The calibration seed set (seed set 1) consisted of 120 seeds obtained from plants of the two ecotypes, Esfahan (non-irrigated and control) and Naeen (control) and the seeds were divided based on colour; yellow, grey and black in accordance with Lucena *et al.* [8]. Images of the whole seeds were captured by the VideometerLab (Videometer A/S, Hørsholm, Denmark) and subsequently each single seed was imbibed and cut for tetrazolium test. Images of the cut seeds were captured before and after immersion in the tetrazolium solution (described below).

The validation seed set (seed set 2) consisted of 4 × 75 seeds obtained from plants of the two ecotypes, Arak and Ahvaz, grown under control and non-irrigated conditions. The seeds were divided in accordance to the colour of the seed coat (yellow, grey and black). The weight of each single seed was determined with four decimals on a SI-234 analytical balance (Denver, Bohemia, NY, USA). Images were captured by the VideometerLab and subsequently a germination test was performed. The non-germinating seeds were immersed in tetrazolium.

### 2.2. Tetrazolium Test

Dry seeds were placed between moist paper towels to maintain moisture for 18 h at 30 °C. After this period, the seed coat was removed and the seeds were cut longitudinally and images were captured. Then seeds were immersed in a tetrazolium solution at 0.1% and kept in an oven at 35 °C in the absence of light for 120 min. Red colouring of the embryo indicates enzyme activity, and seeds were therefore scored as viable and the remaining seeds were considered dead (in accordance to the work by Gaspar-Olivera *et al.* [10]). Images were captured of each single seed after staining with tetrazolium.

### 2.3. Germination Test

Germination was performed between paper, in accordance to ISTA [18]. In each box 25 seeds were placed on wet pleated filter paper and germinated at 25 °C for a 14/10 (light/dark) hour photoperiod. Counting was done every day for two weeks. After completion of the germination test seeds which did not germinate were stained with tetrazolium.

### 2.4. Multispectral Imaging

**Figure 1.**
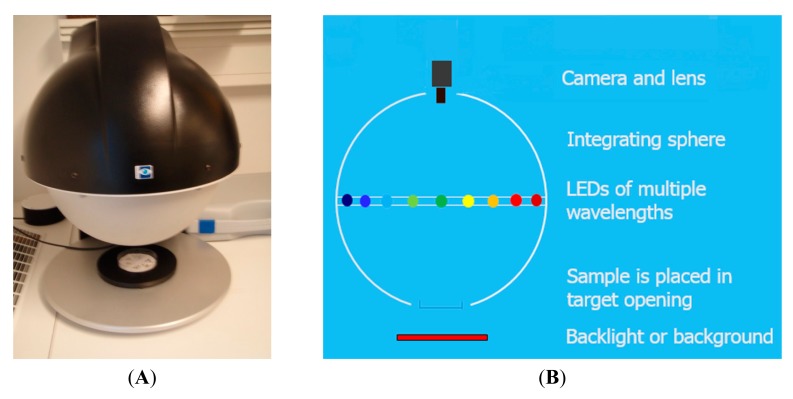
(**A**) Picture of the VideometerLab instrument and (**B**) is the outlines setup of the VideometerLab instrument.

The spectral imaging system used in this research is a VideometerLab instrument (Videometer A/S). It consists of a 5 mega pixel CCD camera, mounted inside the top of an integrating sphere, coated with highly white and diffusing paint and illumination by narrowband high-power LED (light-emitting diodes) placed at the rim, and thereby ensure a uniform and diffuse illumination of the sample at the bottom port of the sphere reflection (Figure 1). The LED's provide light in succession (sequential strobes) at the following 19 wavelengths: 375, 405, 435, 450, 470, 505, 525, 570, 590, 630, 645, 660, 700, 780, 850, 870, 890, 940 and 970 (and backlight at 625 nm beneath the sample holder). Before capturing images the instrument is calibrated to absolute reflectance using a bright and dark reference object (NIST traceable targets), and geometrically aligned using dotted plate. The seed sample is placed at the bottom of the integrating sphere and within 5–10 s a high-resolution multispectral image of 2056 × 2056 pixels were captured. The technology behind the system has originally been developed at the Technical University of Denmark and is described in details in a patent from 2006 [19].

### 2.5. Data Analysis

Data analyses were performed using VideometerLab software version 2.13.73 (Videometer A/S). The multispectral images (MSI) were transformed using normalized canonical discriminant analyses (nCDA) in order to minimize the distance to observations within the seed colour classes and to maximize the distance to observations between classes. The first part of the analysis was to build a mask to segment the seeds from the background, which was based on an nCDA transformation of seeds and filter paper and a simple threshold. Next all seeds were collected in a blob database from which different colour, texture and shape features of the individual seeds could be employed. The colour feature MulticolourMean extract the mean intensity of the reflected light for each single wavelength. It was extracted for the two seed sets (calibration and validation sets), and presented as a mean intensity spectrum. The feature RegionMSImean, calculate a trimmed mean of MSI transformed pixel values within the blob (each single seed). It was extracted from seeds in the two seed sets. In the calibration set (seed set 1) the MSI was based on an nCDA transformation between the 94 seeds that were stained red in the tetrazolium test (viable seeds) and the 26 seeds that remained unstained (dead seeds). The threshold value was set to zero, so negative values correlated for viable seed and positive values correlated for dead seeds. The same MSI transformation was employed to seed set 2 for validation of data (containing 4 × 75 seeds from Arak and Ahvaz, grown under control and non-irrigated conditions respectively).

Extracted data were further handled in Excel, where the RegionMSImean values were correlated to the germination capacity. Differences among reflection spectra were analyzed using linear mixed models (fixed effects: wavelength and seed class (colour or germination capacity); random effect is the replicates) using Rv 3.0.2 (RStudio. ink). Model fits were assessed by visual inspection of residual and normal probability plots.

## 3. Results and Discussion

**Figure 2.**
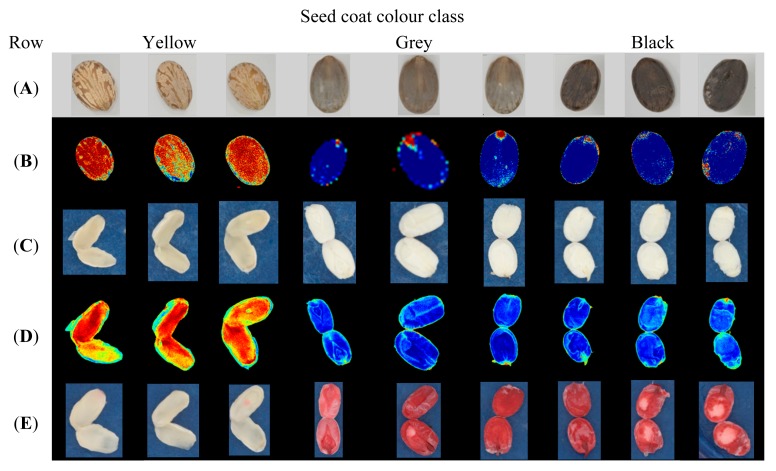
Overview of seeds divided into three classes based on visual colour of seed coat: yellow, grey and black. Row (**A**) shows RGB images of the intact seeds; (**B**) is images transformed by nCDA to divide dead and viable seeds (intact seeds); (**C**) is RGB images of cut seeds; (**D**) is images transformed by nCDA to divide dead and viable seeds (based on cut seeds) and (**E**) is RGB images taken after the cut seeds has been immersed in tetrazolium.

To our knowledge this is the first study on castor, where the seed coat colour reflection has been measured using multispectral imaging and classified in accordance to seed viability. Similar separation of the castor seeds was implemented by Lucena *et al.* [8], based on inspection by the human eye, an evaluation also performed in our study as a primary step (row A, Figure 2). Figure 2 provides an overview of seeds from the ecotype Esfahan, divided into three seed coat colour classes; yellow, grey and black. Visual comparison of the 3 × 3 images of the seeds in row A indicates a variation in colour between all three seed coat colour classes. Row B shows the transformed images of the seeds in row A; here the yellow seeds appear red and the grey and black seeds appear blue. Row C and D display the cut seed and a similar pattern as for the whole seed is observed, demonstrating how transformation of the images, results in red appearance for the cut yellow seeds and blue appearance for the viable cut seeds. Row E displays the result from the tetrazolium staining. The yellow seeds display no staining whereas the grey and black seeds stain the living tissue red, based on dehydrogenase enzyme activity. Thus, our results demonstrate that multispectral imaging can be a non-destructive method in castor seed viability testing as we have observed a satisfying positive correlation between seed coat colour reflection and viability obtained by the tetrazolium test.

**Figure 3.**
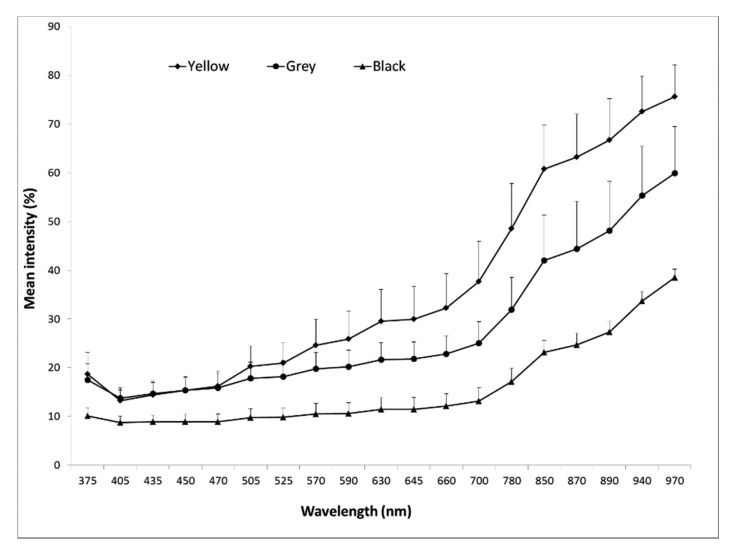
Mean reflectance spectra based on the reflection of the seed coat surface of three seed classes, determined as yellow, grey and black seeds. Vertical bars represent standard deviation (only upper directions bars are shown, to avoid overlapping of bars) from single seed replicates.

The spectra in Figure 3 show the reflection for the three seed classes and from which it can be observed that in the wavelength from 375–470 nm the yellow and grey seeds are alike, but differ when higher wavelength number are employed. The black seeds have lower reflection intensity in comparison to both the yellow and grey seeds in all 19 wavelengths. Statistical analysis based on reflection data from all wavelengths show that the three classes differ significantly. The standard deviation within the classes of black seeds is lower than the variation within the yellow and grey seed classes.

### 3.1. Correlation of Reflection Data with Tetrazolium Test

Table 1 gives an overview of the results from the tetrazolium test and it appears that colour classes could be an indicative selection marker for viability, by choosing grey and black seeds and discarding yellow seeds. In the non-irrigated situation (ecotype Esfahan) it was observed that all nine yellow seeds were dead. Only one out of eight grey seeds were dead (13%) and all the seeds scored as black was viable. For the control seeds (ecotypes Esfahan and Naeen), 11 out of the 16 yellow seed (69%), two out of 35 grey seeds were dead (6%) and three out of 38 black seeds (8%) were dead.

**Figure 4.**
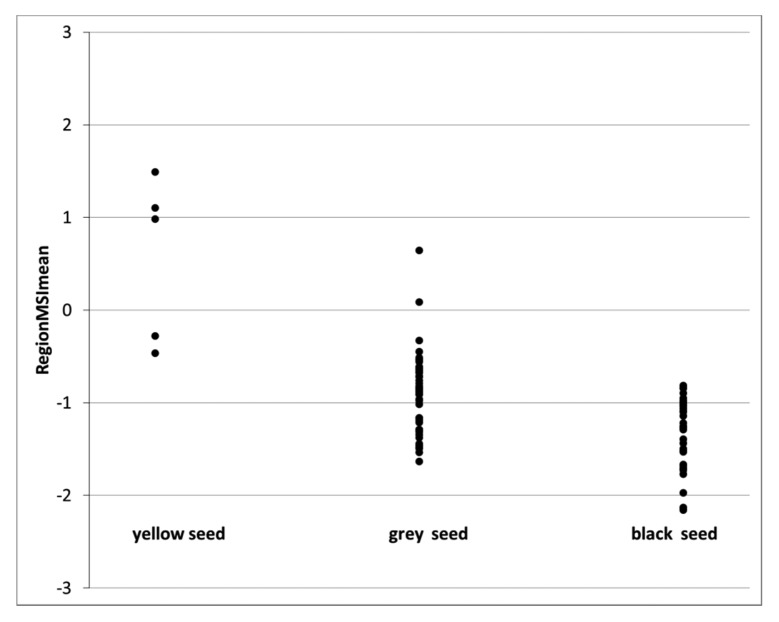
Positive RegionMSI mean values is predicted as dead seeds and negative values predicted as viable seeds for seeds with enzyme activities in the tetrazolium test. Three seed classes divided due to the colour of seed coat (yellow, grey and black).

**Figure 5.**
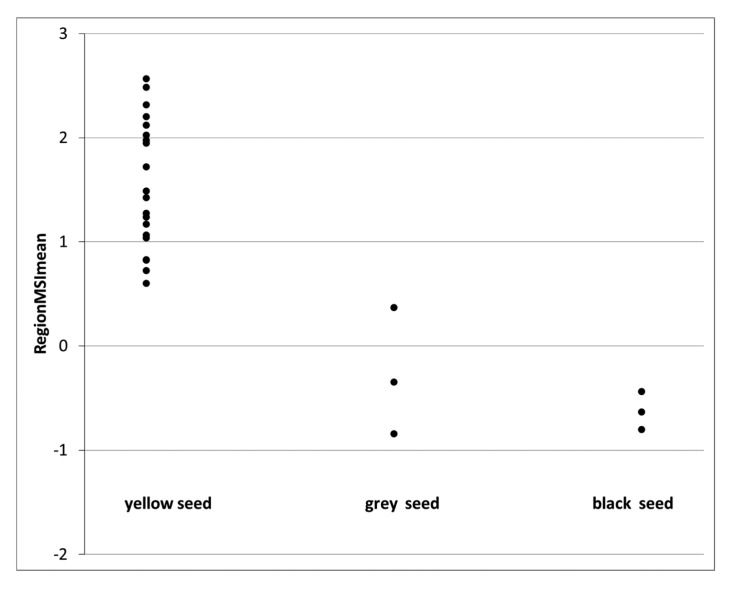
Plot of RegionMSI mean value of the seeds that are physically dead (positive values predicted as dead seeds and negative values predicted as viable seeds) for seed with no enzyme activity in the tetrazolium test (dead seeds). Three seed classes divided according to the colour of the seed coat (grey, black and yellow).

The observations in Table 1 were employed to build a calibration model (based on nCDA transformation between viable and dead seeds) and results from this calibration set are shown in Figures 4 and 5. Figure 4 show the distribution of RegionMSImean values obtained from the viable seeds and it was observed that most of the values were negative and belongs to the seed classes' grey and black. In Figure 5 most values were positive and obtained from seeds determined yellow. In total 94 seeds were determined as viable according to the tetrazolium test and according to the RegionMSImean feature 89 seeds had a negative value and were classified as viable. In the calibration set 26 seeds were determined as dead according to the tetrazolium test and according to the RegionMSImean feature 21 seeds were classified as dead. In the calibration set 92% of the 120 seeds were correctly classified (when zero was set as the threshold value) (Table 2).

### 3.2. Prediction of Germination

**Figure 6.**
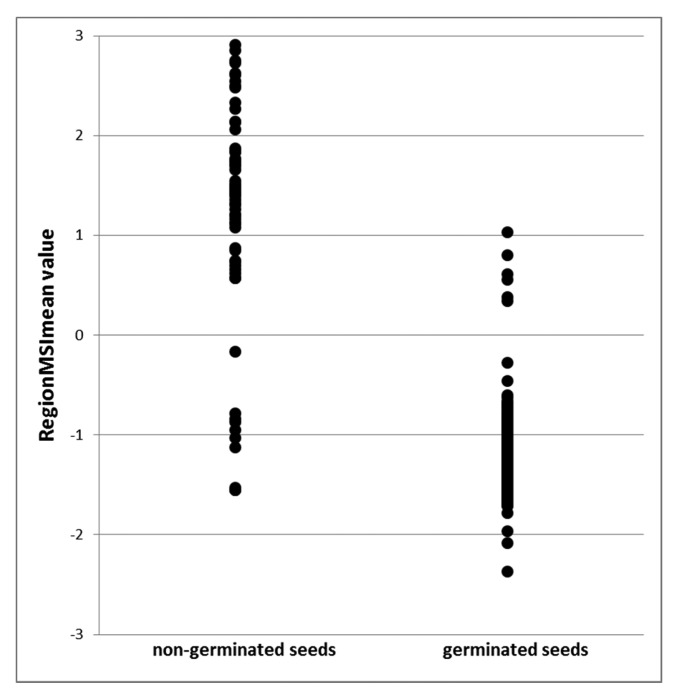
Positive RegionMSI meanvalues is predicted in the model as dead seeds and negative values predicted in the model as viable seeds. Seeds evaluated for germination after 6 days.

**Figure 7.**
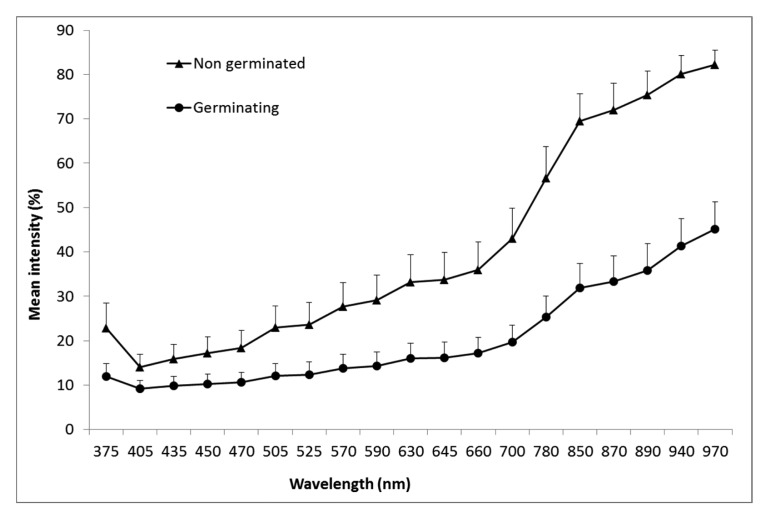
Mean reflectance spectra based on the reflection from the seed coat surface of seeds that germinate and non germinating seeds. Vertical bars represent standard deviation (in upper direction) from single seed replicates.

Next step was to evaluate how this RegionMSImean feature, using the MSI transformation from seed set 1 (calibration set) could be valuable in predicting the seed germination capacity. The extracted feature values for each single seed are plotted in Figure 6. For the non-germinating seeds, most of the data points are positive indicating that the seeds were dead and not able to germinate. At the opposite end of the scale, most of the points has a negative value and could be predicted as germinating seeds. In Table 3 it was observed that 241 seeds were predicted as viable and 231 seeds germinated. Five out of the 10 non-germinating seeds were determined as dead in the tetrazolium test. 59 seeds were predicted as dead, in the nCDA model and six of these germinated and the rest (53 seeds) were also determined dead in a tetrazolium test. In total 96% of the seeds were correct classified. Evaluating of the spectral reflectance it was observed in Figure 7 and by statically analysis, that there were a significant different between the mean intensity value for germinating and non–germinating seeds, and with the best separation using the near infrared reflecting (NIR) wavelengths. The important of the NIR wavelength in prediction of germination capacity has been highlighted in earlier vision studies, where multispectral imaging was tested on spinach seeds [11].

**Figure 8.**
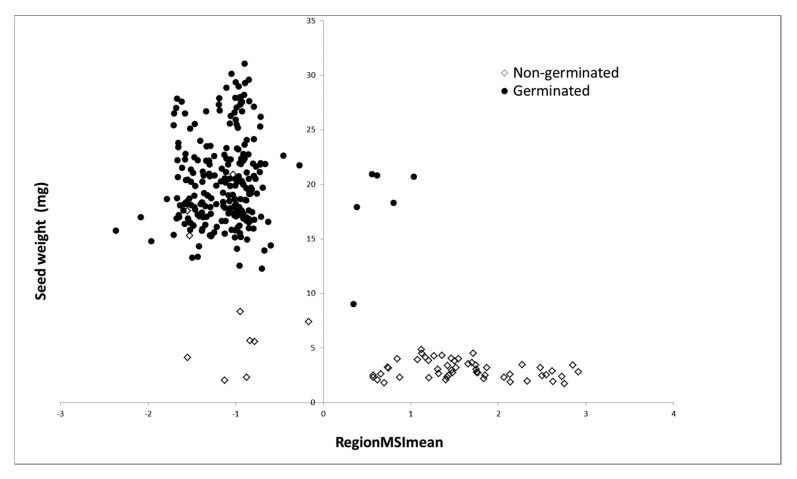
Plot of seed weight and RegionMSImean value (feature extracted from images) for germinated and non-germinated seeds. Seeds below zero are predicted as viable and seeds above are predicted ad dead seeds.

In previous studies seed filling and thus seed weight has been mentioned as a predictor for maturity and germination capacity [6]. Severino and Auld [5] found that castor seeds below 0.16 g had an average germination percentage of 0.1%. Similar result is obtained in our studies, where most of the tested seeds below 0.15 g did not germinate after 14 days of germination. In our study germinating seeds ranged from 0.1–0.35 g, which was considered as low weight in the above mentioned studies where the seed weight were up to 0.90 g. The difference in seed weight between the two studies can be due to different ecotypes or dry matter percentage of the seed samples, at the time of measuring. The correlation between seed coat colour and germination capacity can be due to the different maturity stages at the time of seed harvest. It was observed in Figure 8 that most of the seeds with a low weight also display a positive RegionMSImean (yellow immature seeds). The use of seed coat colour as a marker for maturity has also been proven in other species. The epidermis layer of *Arabidopsis* seed coat contains phenol compounds, which is colourless until last stage of development, where it becomes dark brown [20,21]. In *Brassica* species the chlorophyll content in testa is degraded during maturation [22]. In our study we highlighted that five of the yellow seeds were viable and six of the grey/black seed were dead, which indicate that a more detailed subdivision of the seeds might be needed. Greenwood and Bewley [23] have proposed three major physiological periods of seed development of castor bean: (1) the period of rapid fresh weight; (2) the period of dry weight gain and (3) the period of maturation. These three periods may be distinguished by testa colour alone. In their study they have further subdivided each period into several stages and highlight that these cannot be distinguished using a single morphological trait. Depending on the development stage the seeds may also exhibit primary dormancy [24]. During storage deterioration and membrane damage might occur and biochemical changes have been determined in earlier studies [25,26]. This adds to the explanation of why ten seed were classified as viable, but failed to germinate (Table 3). Thus, a combination of our analysis with technologies providing information from inside the seed, like X-ray, NIR or infrared reflection (IR) would be valuable.

## 4. Conclusions

The results suggest that reflection data from the castor seed coat can be valuable in prediction of seed viability. All seeds that visually were sorted as yellow could be determined as dead in the tetrazolium test and a variation of viability was observed within the grey and black seeds. A supervised classification model, based on nCDA transformation between viable and dead seeds, was built and tested on a new set of seeds. Here the prediction of viable and dead seeds resulted in 96% correct classified seeds, and confirm the potential of using multispectral vision technology in seed quality testing. The benefit of using multispectral imaging in comparison to instruments such as single seed NIR or IR, is the possibility to measure the multiple components by reflection from both visual and NIR wavelengths. Hence information of many valuable seed quality traits can be extracted in one single measurement e.g., seed size, colour, viability, physical purity and seed health (if pathogens are visual on the surface of the seed coat).

## Figures and Tables

**Table 1.** Overview of seed material employed in tetrazolium test and for development of calibration model.

**Seed Material**	**Colour Classes**	**Number of Seeds**	**Result from Tetrazolium Test**	
	
		**Total**	**Viable**	**Dead**
Esfahan non-irrigated	Yellow	9	0	9
	Grey	8	7	1
	Black	24	24	0

Esfahan Control	Yellow	8	5	3
	Grey	11	11	0
	Black	16	13	3

Naeen Control	Yellow	8	0	8
	Grey	24	22	2
	Black	12	12	0

**Table 2.** Data from calibration set (seed set 1). The number of viable and dead seeds observed in the tetrazolium test and the number of seeds classified as dead and viable in the calibration model, based on an nCDA transformation employed in the Region MSI feature.

	**Seeds Classified as Viable**	**Seeds Classified as Dead**	**Seeds in Total**
Viable in tetrazolium test	89	5	94
Dead in tetrazolium test	5	21	26

**Table 3.** Data from the validation set. Number of seeds classified as viable and dead and germination results after six days of germination. The number in brackets shows the result after a tetrazolium test of the non-germinated seeds. The number of seeds classified as dead and viable in the prediction model, were based on an nCDA transformation employed in the Region MSI feature.

	**Seeds Classified as Viable**	**Seeds Classified as Dead**	**Seeds in Total**
Germinated	231	6	237
Non-germinated	10 (5 viable and 5 dead)	53 (53 dead)	63
